# Design and Manufacturing of a Nd-Doped Phosphate Glass-Based Jewel

**DOI:** 10.3390/ma13102321

**Published:** 2020-05-18

**Authors:** Diego Pugliese, Federico Simone Gobber, Ilaria Forno, Daniel Milanese, Marco Actis Grande

**Affiliations:** 1 Department of Applied Science and Technology (DISAT), Politecnico di Torino, Corso Duca degli Abruzzi 24, 10129 Torino, Italy; federico.gobber@polito.it (F.S.G.); marco.actis@polito.it (M.A.G.); 2 Consorzio Interuniversitario Nazionale per la Scienza e Tecnologia dei Materiali (INSTM), Via G. Giusti 9, 50121 Firenze, Italy; daniel.milanese@unipr.it; 3 BMC Gioielli, Circonvallazione Ovest Zona CO.IN.OR Lotto 3A, 15048 Valenza (AL), Italy; ilaria.forno@bmcgioielli.com; 4 Department of Engineering and Architecture, Università di Parma, Parco Area delle Scienze 181/A, 43124 Parma, Italy

**Keywords:** jewelry design and manufacturing, phosphate glass, Neodymium, Ashby’s map

## Abstract

This paper reports the results of the designing, manufacturing and characterization of a jewel obtained by means of coupling the dogmas of industrial design to the analytical engineering approach. The key role in the design of the jewel was played by an in-house synthesized Neodymium (Nd)-doped phosphate glass, selected due to its easy handling and capability to change color according to the incident light wavelength. The glass core was covered by a metal alloy to mitigate its relatively high fragility and sensitivity to thermal shock and, at the same time, to highlight and preserve its beauty. The selection of the proper metal alloy, having thermo-mechanical properties compatible with those exhibited by the glass, was carried out by means of Ashby’s maps, a powerful tool commonly adopted in the field of industrial design.

## 1. Introduction

Jewelry design is a complex process, which might take into account several different perspectives [1,2,3,4,5,6]. While artists are generally mainly focused on the true essence of design, intended as the coherence of the object as the expression of the designer’s intent, technicians tend to approach it as a sequence of steps targeted at the prevention/solution of production issues directly related to manufacturing.

In the last decades, an always growing approach towards the innovation in design has been adopted in the use of digital technologies, applied both in terms of computer software and prototyping [7,8,9,10,11,12,13]. Editable computer-aided drafting (CAD) representations, accurately capturing the original design and allowing re-parameterization and modification prior to manufacturing, have been developed and studied [14]. Semantic feature modeling systems for sketch-based jewelry design were also proposed [15]. The success of a jewel, however, may not only be related to its design but, more and more, also to the choice of its constituting material/materials, normally dictated by the design and meeting its product requirements [16,17].

Nevertheless, this is not the only possible way. Sometimes new products simply derive from “innovative” or “advanced” materials or even “tailored” ones. Innovation in materials inspires creativity, as much as it boosts technical design. Materials may therefore be a source of inspiration for product designers, suggesting novel visual, tactile, sculptural and spatial solutions to product design. Technical design considers materials as a resource and as a medium. Materials are identified by their properties, and a great part of the game consists in playing with these properties, tuning them in order to optimize both the final product and the way to manufacture it [18]. The tailoring of properties is particularly applicable to gemstones, so as to tune the optical properties, as reported in D. Bootkul et al. [19]. 

Designers are often interested in a wider set of features. According to this approach, they usually describe materials in a qualitative way, appearing bewilderingly vague to technicians. It is therefore essential to establish communication, thus deepening the understanding of the requirements of each phase [20,21].

This becomes even more crucial when considering jewelry design, due to the emotional value of jewels related to social, cultural and historical dynamics. A jewel is deeply linked to memories, emotions and symbols, and it is therefore able to give rise to instinctive reactions [22,23]. 

The present work reports the study of a pendent designed and produced by coupling the dogmas of industrial design to the analytical approach typical of engineering. The lead role in the design of the jewel was played by a specific phosphate glass, originally designed and developed for application in optical fibers through the use of rare-earth (RE) ions doping [24]. 

This latter procedure is commonly adopted to get peculiar properties in laser optics. Among the different RE dopant ions, the Nd^3+^ ion is one of the most important activators for crystalline and bulk glass lasers, thanks to the power and efficiency available from the Nd^3+^: ^4^F_3/2_ → ^4^I_11/2_ radiative transition which causes the emission of near-infrared (NIR) light at the wavelength of around 1.06 μm [25,26]. Neodymium has been extensively used for the development of glass-based lasers and finds applications in a variety of fields, ranging from low-power marking lasers to high-power lasers for material processing [27]. 

Up to now, most of the research activity on fiber lasers has been performed on doped silicate glasses, thanks to their outstanding properties, in particular their extremely low propagation losses, the possibility to withstand high temperatures, and their impressive mechanical strength in tension and even in bending [28]. However, silicate glasses also present some drawbacks: for example, RE ions display a very low solubility in this glass system, and they are prone to clustering [29].

In recent years, phosphate glasses have demonstrated to be a promising alternative to silicate glasses as a host material, especially for high-power applications. In fact, they allow extremely high doping levels of RE ions and thus the fabrication of more compact and active devices [30,31]. The phosphate glasses are very well known for their suitable mechanical and chemical properties, homogeneity, good thermal stability and excellent optical properties [31,32]. If compared to silicate glasses, they exhibit low glass transition (***T**_**g**_*) (400–700 °C) and softening (***T**_**s**_*) (500–800 °C) temperatures, which facilitate their processing and fabrication by melt-quenching technique [33,34]. Being intrinsically highly hygroscopic, thus influencing the effect of RE doping, their water content must be controlled to be as low as possible. Phosphate glasses are generally interesting to be used as biomaterials because of their ability to dissolve completely in aqueous solutions into safe dissolutions, commonly found in the human body [35]. Another important feature of phosphate glasses is their thermal and mechanical strength, which allow the production of optical fibers that can be cleaved and fusion-spliced with commercial optical fiber components based on silicate glasses, thus allowing an easier integration of these fibers in commercial systems [36].

In the present study, the decision to assign the key role in the jewelry design to a phosphate glass was dictated by a double aspect, namely its greater solubility of RE ions and its easier processing with respect to a silicate glass. Moreover, Nd^3+^ was selected as the doping ion for a purely aesthetic purpose, providing its ability to give the jewel a variety of different colors ranging from pink to violet depending on the wavelength of the light used to illuminate it. The research activity carried out to get the specific Nd-doped phosphate glass passed through different steps: at first a passive, stable, robust phosphate glass host able to incorporate high amounts of RE ions was obtained. The glass was then doped with a suitably high Nd^3+^ ions concentration, to confer an intense pink/violet coloration to the jewel.

Finally, in order to limit the fragility and sensitivity to thermal shock typical of a glass and at the same time to highlight and preserve its beauty, the design concept became a glass core covered by a metal alloy, which was properly selected by means of Ashby’s maps. In this aspect, the applied logic was to identify an alloy showing (mainly thermal) properties compatible with those of the proposed glass.

## 2. Experimental Details

### 2.1. Glass Fabrication and Characterization

The glass sample used in this work was synthesized by the conventional melt-quenching technique using chemicals (P_2_O_5_-K_2_O-Al_2_O_3_-BaO-PbO-La_2_O_3_) with high purity level (>99%). The glass host was doped with 0.6 mol% Nd_2_O_3_, corresponding to a Nd^3+^ ions concentration equal to 1.6 × 10^20^ ions/cm^3^. 

The chemicals were weighed and mixed within a dry box under dried air atmosphere, then transferred into an alumina crucible and melted in a vertical furnace (14411, Pro.Ba. Srl, Cambiano (TO), Italy) at 1400 °C for 1 h. A mix of O_2_/N_2_ gases was purged into the furnace during the melting in order to minimize the hydroxyl ions (OH^−^) content in the glass. The melt was cast into a preheated ad-hoc designed brass mold, then annealed at a temperature around the glass transition temperature, ***T**_**g**_*, for 5 h to relieve glass internal stresses, and finally cooled down slowly to room temperature.

A second batch of glass, undergoing the same processing, was cast into a rod 12 mm in diameter to be used for optical, spectroscopic and thermal characterization. In detail, a slice of this rod was optically polished to a thickness of 1 mm and employed for refractive index and ultraviolet-visible-near infrared (UV-Vis-NIR) spectroscopy measurements. Samples with thicknesses of 12 and 5 mm were used for density and coefficient of thermal expansion (CTE) measurements, respectively, and about 200 mg of fine grain sample was prepared and used for the evaluation of the glass characteristic temperatures.

The density of the glass was measured at room temperature by Archimedes’ method, using distilled water as the immersion fluid. The Nd^3+^ ions concentration was calculated through density data in relation to the nominal composition of the glass.

The characteristic temperatures of the glass (glass transition temperature ***T**_**g**_* and onset crystallization temperature ***T**_**x**_*) were measured using a differential thermal analyzer (DTA 404 PC Ёos, Netzsch-Gerätebau GmbH, Selb, Germany) with a heating rate of 5 °C/min in sealed Pt/Rh pans. An error of ±3 °C was observed in measuring the characteristic temperatures.

The coefficient of thermal expansion (CTE) was measured by a horizontal alumina dilatometer (DIL 402 PC, Netzsch-Gerätebau GmbH, Selb, Germany) operating at 5 °C/min. The measure was automatically interrupted when a shrinkage higher than 0.13% was reached (softening point ***T**_**s**_*). The CTE value was calculated in the 200–400 °C temperature range. 

The refractive index of the glass was measured in the visible region at 633 nm by a prism coupling technique (2010, Metricon Corporation, Pennington, NJ, USA). Ten scans were performed for each measurement and the estimated error of the measurement was ±0.001.

The absorption spectrum of the glass was measured at room temperature for wavelengths ranging from 300 to 900 nm using a double beam scanning spectrophotometer (UV-2600, Shimadzu, Columbia, MD, USA).

### 2.2. Jewel Design and Manufacturing

The jewel, specifically a pendent, was designed to be made of bezels fabricated by direct casting. Finishing and assembly of the jewel consisted of traditional operations. Since finishing entailed tumbling and polishing, particular care was devoted to the choice of the optimal process parameters that would not damage the metal shape. The desired geometry of the glass beads led to the need for specific casting molds. Phosphate glasses can be easily cast into copper molds, resulting in highly glossy surfaces. The required mold was then designed taking into account the metal and glass shrinkages and setting the appropriate tolerances. The cavities were first rapid prototyped, then cast in 98Cu2Be (wt%) alloy and finally assembled using an aluminum frame to obtain a mold with multiple cavities in order to maximize the number of glass beads that can be cast at each cycle, considering the narrow temperature range in which the glass material provides the proper viscosity to be poured correctly.

The cast glass inserts were then ground to obtain a flat-bottom surface with a matte finish in order to enhance the perception of color change, since the light passing through the glass would be absorbed and diffused in the matte layer. Flat metal frames were manufactured by directly casting a Fusia 444 resin (DWS, Thiene (VI), Italy), and following an optimized production process detailed in [37], with 36:100 water/powder ratio on Plasticast^®^ investment (Ransom & Randolph, Maumee, OH, USA) and subsequent burnout in a ventilated kiln. A special mold was required to inject the wax on the glass inserts employing the same technique used to perform the hollow casting [38].

**Figure 1:**
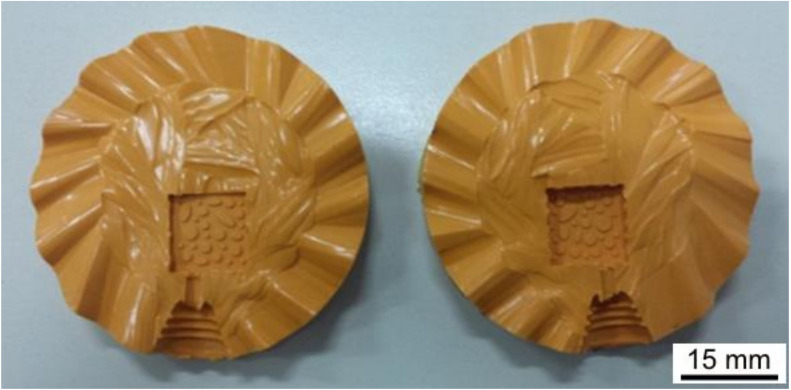
Silicone rubber mold for the metal bezel.

A resin model, representing the glass cab with the metal bezel surrounding it, was grown using the rapid prototype technique. A silicone rubber mold was made on this model and it was cut open in a traditional way (Figure 1).

After the resin model was removed from the mold, one glass cab was placed in the mold and the wax was injected around it forming the bezel. After investing and burnout, the metal could be cast directly on the glass creating an element that could later be assembled by modern welding and soldering techniques. Parts were cast in the selected metal alloy in a vertical casting equipment (TVC-10, Topcast, Monte San Savino (AR), Italy) with a graphite crucible and argon as protective gas.

The selection of a suitable metal alloy followed different requirements, starting from the need to meet both technical and aesthetic issues. Another pre-requisite was related to the joining of the glass and the metal, so as to make the former material able to withstand the casting phase and preserving the optical characteristics even after exposure to the high temperatures typical of the investment burnout. From a technical viewpoint, the metal alloy was required to show low viscosity when molten to fill thin sections and to exhibit a solidification shrinkage similar to the one of the glass. Moreover, it had to be easy to cast at relatively low temperatures to avoid damage to the glass, to polish with low abrasive tools and to weld and/or braze. It is clear how the selection of the vitreous material highly influenced the choice of the metal. In order to identify materials with matching properties, several different methods and instruments can be employed. In the specific case of this study, the proper combination of metal and glass to manufacture the jewel was selected by using Ashby’s maps [16,18].

The first analysis was focused on the linear thermal expansion coefficient. The coefficients of the selected glass and metallic alloy had to be very similar to avoid excessive thermal stresses on the glass core. Moreover, the melting point of the alloy had also to be taken into account to avoid damage to the glass insert. 

**Figure 2:**
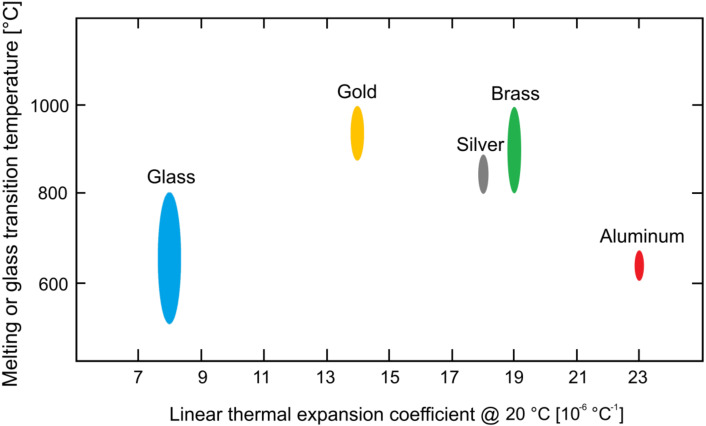
Ashby’s map of the melting point or glass transition temperature vs. linear thermal expansion coefficient of the materials under consideration for the manufacturing of the jewel [39].

A dedicated Ashby’s map is plotted in Figure 2 in view of giving a visual understanding of the material properties to drive the choice.

The analysis of the Ashby’s chart reported for the linear thermal expansion coefficient highlights that the silver alloys appear to be the best candidates for the fabrication of the jewel. In particular, their casting temperature allows the glass to be heated with the investment to a normal curing temperature, thus avoiding the exposure to levels which are not compatible with the material’s resistance. 

Conversely platinum, palladium, gold and brass alloys are not compatible with the selected glass due to their higher melting ranges (Pt- and Pd-based alloys show melting ranges above 1200 °C), while aluminum alloys are so far not considered for jewelry production mainly given their low density, which is generally perceived as related to a poor quality product.

An alloy based on the Ag-Cu system (composition is reported in Table 1) was identified as matching the requisites. The alloy was also chosen due to its superior corrosion resistance if compared to conventional Ag 925 sterling alloys, specifically against tarnishing [40]. Nickel concentration in the alloy is very low and, after laboratory tests, the release rate of this element was confirmed to be 0.004 µg/cm^2^/week, far below the 0.2 µg/cm^2^/week lower limit imposed by the European standards [41].

The casting temperature of the metal was accurately identified in order to prevent or at least reduce the thermal shocks and diffusive phenomena between the metal and the glass. This revealed to be of paramount importance in view of avoiding damage to the glass inserts during the casting phase.

Prior to casting, the burnout cycle of the investment was modified for the purpose of shortening the length at the peak temperature and keeping the casting temperature of the mold as low as possible to avoid diffusion between the glass and the silica in the investment.

The final mold temperature was designed to be a compromise between two fundamental constraints. The first one is related to the casting requirement of a high enough temperature for the molten metal to fill all the cavities before starting the solidification process. The second one is related to the preservation of the inner glass core, but also to the need of reaching a mold temperature high enough to reduce the thermal stresses due to the contact with the molten metal, while at the same time low enough to prevent the softening of the glass and the diffusion into the investment.

### 2.3. Jewel and Alloy Characterization

In order to identify the most accurate melting temperature, a differential scanning calorimetry (DSC) analysis was performed on the Ag-based alloy with a thermal analyzer (mod. 92-16.18, Setaram, Caluire, France). Samples were placed in an Al_2_O_3_ crucible and then heated, in inert Ar fluxing atmosphere, up to 1000 °C at 20 °C/min heating rate to detect endo/exo peaks associated with phase transformations. 

After production of the metal-glass jewel, the characterization of the final product was performed to assess the effectiveness of the design/manufacturing approach. A sample was obtained from the jewel by cutting it in half by means of an abrasive disc and then it was mounted in a phenolic resin. The sample was gently ground and then polished with cloths and diamond suspensions (6, 3, 1 µm). The final super finishing treatment on the cut surface was done by polishing with colloidal silica.

Microstructural, compositional and mechanical properties of the alloy were evaluated, while the embedded glass was analyzed by Raman spectroscopy to assess any possible modification due to the thermal alteration when casting the silver-based alloy. The microstructural analysis was performed by scanning electron microscopy (SEM) observation (mod. Leo 1450 VP, Zeiss, Oberkochen, Germany) and the composition of the alloy was verified by energy dispersive spectroscopy (EDS) analysis (mod. Oxford 7195 Link Pentafet, Oxford Instruments, Abingdon-on-Thames, UK). The aim of the metallographic analysis was to characterize the microstructure of the alloy and define the metal to glass interaction at the interface. Mechanical properties of the alloy were evaluated by Vickers microhardness (mod. Leica VMHT, Leica AG, Wetzlar, Germany) after ASTM E384 standard; the load of 0.1 kgf was applied for 15 s and then the indentation diagonals were measured. A total of 10 repetitions were made to minimize the experimental error. Raman spectroscopy was performed with a Raman microscope (mod. InVia, Renishaw, Wotton-under-Edge, UK) in a range between 200 and 1450 cm^−1^ for a 532 nm laser. The same glass was analyzed before and after being embedded in the metallic cage in order to ascertain any possible alteration to the glass structure itself related to the input of thermal energy during casting. Peaks indexing was performed after comparing the experimental results to selected literature regarding phosphate glasses [42].

## 3. Results and Discussion

**Figure 3:**
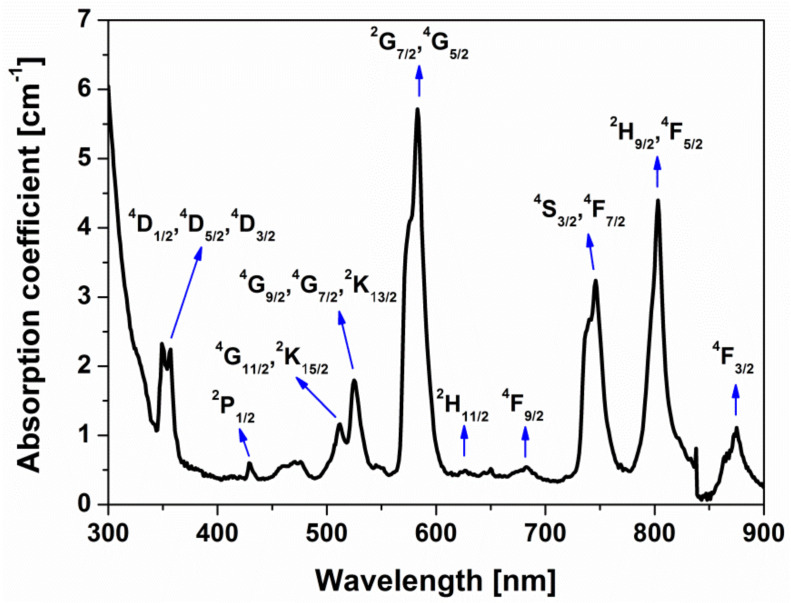
Absorption spectrum of the Nd-doped phosphate glass. The main Nd^3+^ levels are labeled, considering absorption from the ground state ^4^I_9/2_.

The jewel described in this study was fabricated by combining traditional production processes, i.e. casting and polishing, with advanced production ones, consisting in the direct casting of a cage surrounding the glass. As aforementioned, the key role in the design of the pendent was played by a specific Nd-doped phosphate glass, whose main physical, thermal and optical properties are summarized in Table 2 and UV-Visible-NIR absorption spectrum is reported in Figure 3.

The glass exhibits several inhomogeneously broadened bands in the near-UV, Vis and NIR regions, which are assigned to the transitions from the ground state ^4^I_9/2_ to the excited states of Nd^3+^ ions.

**Figure 4:**
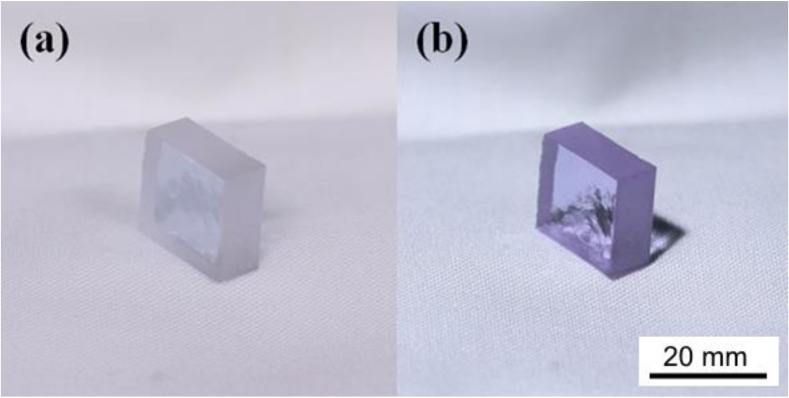
(**a**) As-cast Nd-doped phosphate glass illuminated by white neon light and (**b**) visible light spectrum.

The glass was selected due to its fascinating optical properties, specifically its ability to show a great variety of pink/violet shades according to the illumination source. It appeared pink when exposed to the cold light of offices (Figure 4a) and turned to be violet when illuminated by sunlight (Figure 4b).

Moreover, the glass presents the advantage of being easily cast in simple shapes using copper-based molds, and shaped by grinding and polished. Conversely, it is worthwhile underlining that it is a relatively fragile and thermal shock sensitive material, therefore the use of only glass was clearly not suitable neither for the aesthetic aspect nor for the technical one.

In order to highlight and preserve the beauty of the glass, the design concept became a glass core surrounded by metal frames. The interface between the glass inserts and the metal frames provides an ideal example of the benefits derived when designers (art) and engineers (science) collaborate. 

**Figure 5:**
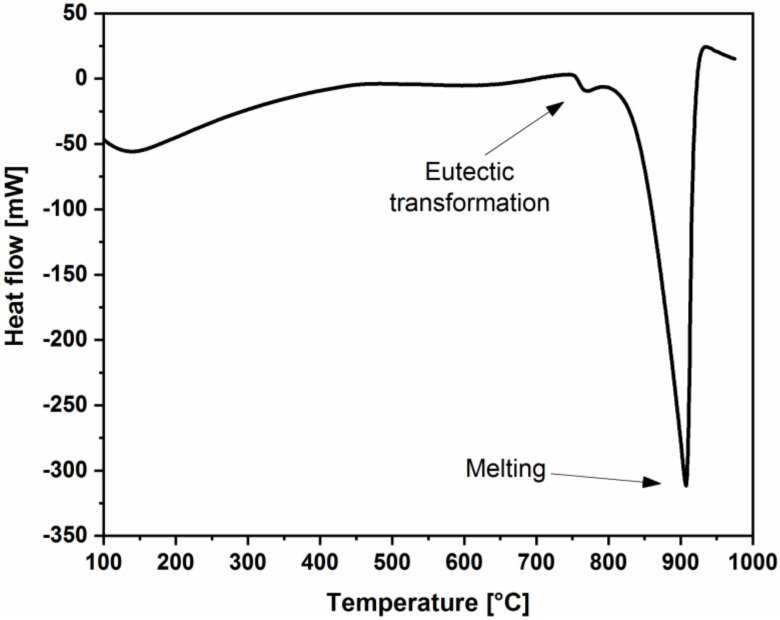
Differential scanning calorimetry (DSC) analysis of the Ag-Cu-Ni-Zn alloy.

Dealing with the metal alloy, according to the results of the DSC analysis, the endothermic peak related to melting occurs at the temperature of 907.68 °C (Figure 5). Based on this value, all the casting parameters were tuned and set. When coupling glass and metal, it is of paramount importance to achieve a proper balance in the choice of temperatures.

On one hand, good fluidity must be guaranteed to the alloy when entering the mold to prevent the melt from freezing; such event would hinder the correct filling of the mold. On the other hand, a too high temperature would damage the glass, thus altering its optical properties. 

**Figure 6:**
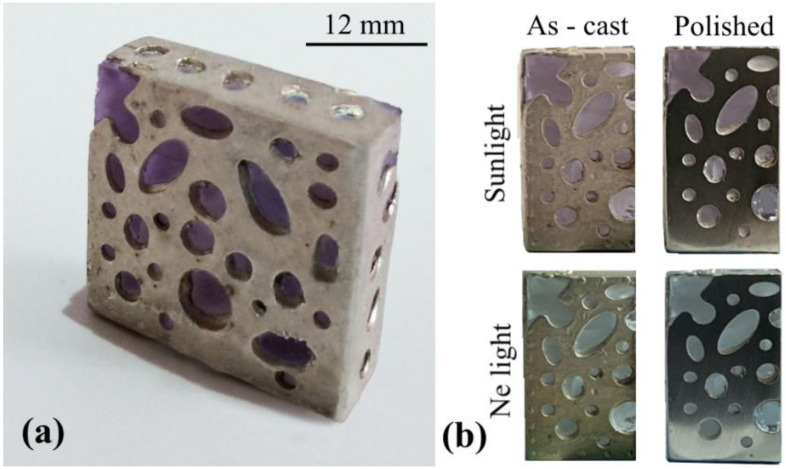
Macrograph of the jewel produced by casting of the silver sterling alloy around the phosphate glass core: (**a**) macrograph of the whole jewel taken in natural lighting conditions, and (**b**) half sections of the jewel in different lighting conditions, before and after surface polishing. The total volume of the pendent is approximately 3100 mm^3^.

A 100 °C superheat was set to guarantee good fluidity and promote the complete filling of the mold. The result obtained after casting of the silver alloy is the object of Figure 6. A continuous metallic frame with round shaped apertures (both oval and circular) surrounds the glass core. The metallic cage reproduces the geometries that were printed in the silicone rubber mold with a high detail of fidelity. The upper-left corner in Figure 6 was deliberately left uncovered from the alloy for placing the ring for hooking the jewel to a necklace. Soon after casting, only surface finishing operations are required before the jewel is ready to wear on a necklace.

At this stage, the choice of the Ag-Cu-Ni-Zn alloy seems to be proper from both the points of view of aesthetics and from that of functionality.

The measured hardness was 81 ± 3 HV_0.1; such value is higher if compared to conventional sterling silver alloys (Ag 925) which stands around 50 HV for the alloy in the as-cast state [43]. As for the literature, the sterling silver-based alloys are heat treatable by a conventional solubilization at 750 °C for one hour with water quenching, followed by an aging process of several hours performed in the 150–350 °C temperature range [43]. The aim of the heat treatment is to dissolve the coarse intermetallic compounds present in the as-cast microstructure with the solubilization plus water quenching and then to promote the precipitation of finer intermetallic compounds having reinforcing properties with the aging treatment. 

The combination of a glass and a metallic alloy in the jewel imposes certain restrictions to this approach; in fact the quenching part of the solubilization would not be withstandable for glass that would be highly prone to cracking. Thus, having an alloy with a higher level of hardness in the as-cast state is a desirable feature. 

**Figure 7:**
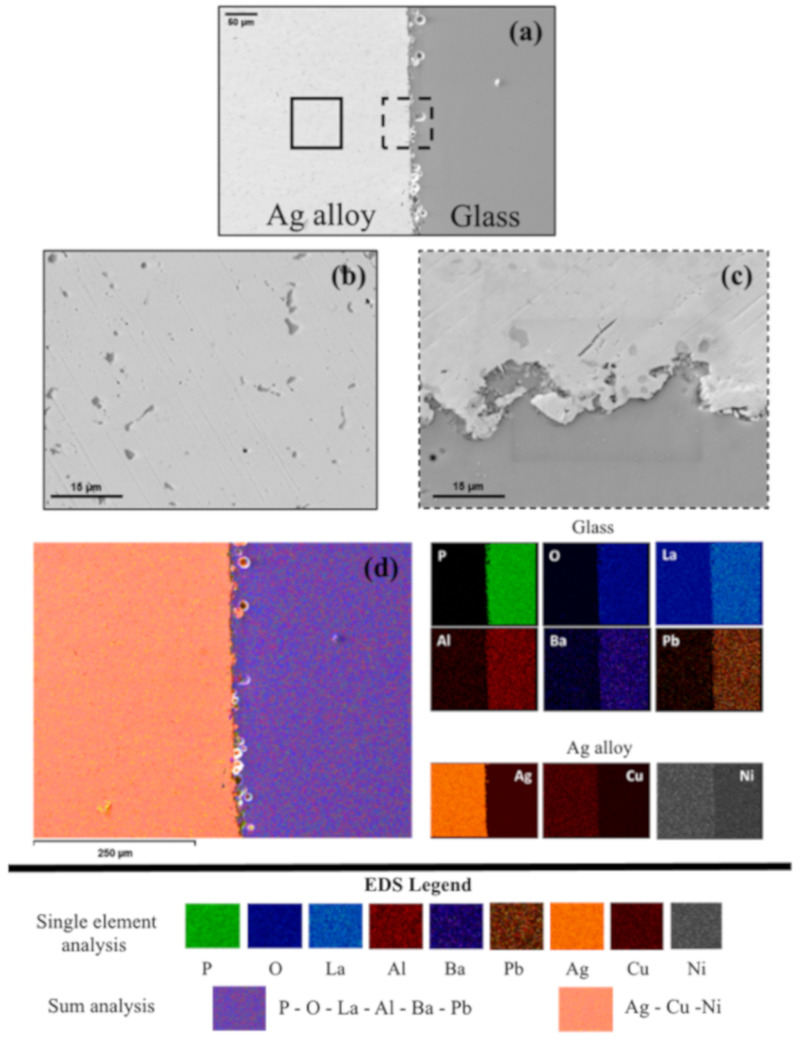
Cross-sectional scanning electron microscopy (SEM) images of the glass/metal interface: (**a**) metal/glass interface at low magnification, (**b**) Ag alloy, (**c**) metal/glass interface at higher magnification, and (**d**) EDS compositional map of the metal/glass interface.

A closer observation of the metal/glass interface by scanning electron microscopy (Figure 7) highlights a continuous profile between the two materials. Some porosities are detectable in the most superficial part of the glass (Figure 7a) but they do not seem to negatively affect the contact between the two materials. Both open and closed porosities are present depending on their location in the glass: pores located at the surface are open while those below it are closed. The silver alloy is biphasic (Figure 7b) with dark Cu-enriched eutectic particles dispersed in the light colored Ag-based matrix [44]. The composition of the two phases was analyzed by EDS and is reported in Table 3.

By increasing the magnification in the interfacial zone (Figure 7c), a good interconnection between the metal alloy and the glass is detectable. The silver alloy, after melting, was poured into the mold where the phosphate glass had been previously placed and was forced to fill the gap between the glass and the mold itself.

The low viscosity of the molten alloy allows both good wetting on the glass surface and the penetration of the alloy inside the open porosities too, thus enhancing the interlocking effect between the two materials (Figure 7c). Another evidence of the penetration of the alloy in the open porosities and the pure mechanical interlocking between glass and metal is given in Figure 7d thanks to the EDS analysis. 

**Figure 8:**
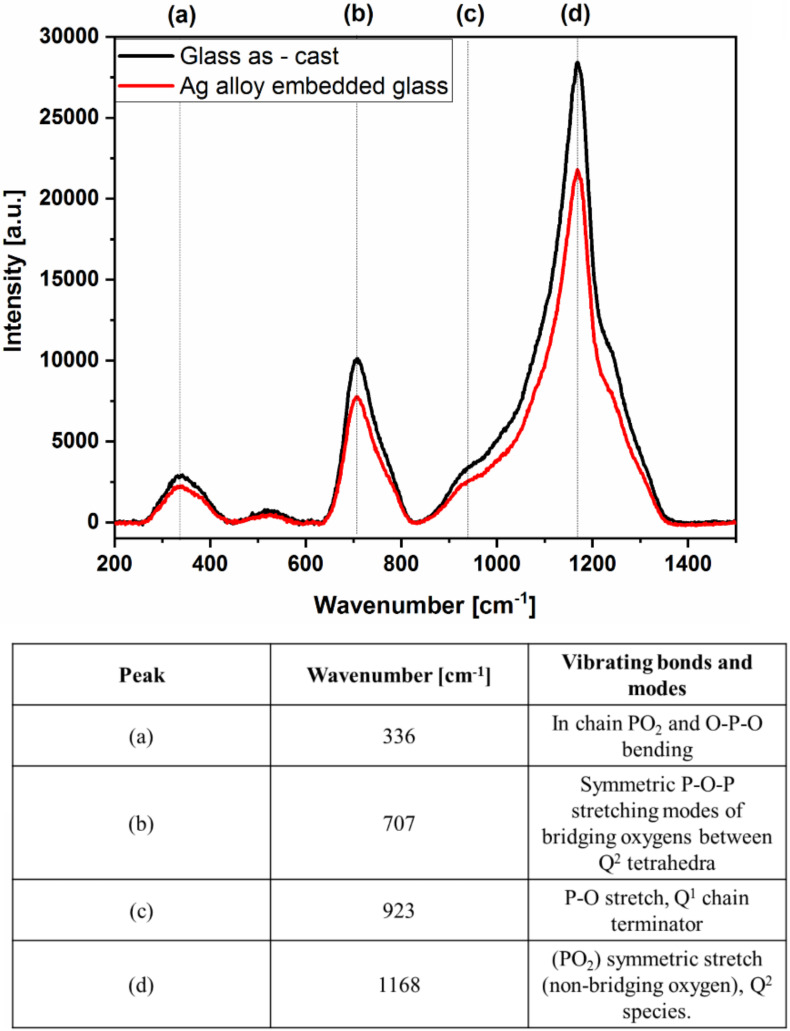
Raman spectra and corresponding peaks indexing for the phosphate glass before and after casting the silver alloy around it.

The constitutive elements of the two materials are separated in the two materials and the contact between the molten alloy and the glass does not result in a chemical interaction between the two. No traces of elements from the glass are detected in the metal alloy, while some traces of the alloy constitutive elements are detected in the open porosities of the glass. This observation confirms the hypothesis that adhesion between the glass and the silver alloy is due to pure mechanical interlocking without the formation of any compound at the interface. No systematic change in the Raman spectra was observed by comparing the spectra of the glass before and after being surrounded by the silver alloy cage (Figure 8). In this latter case, the spectrum was acquired at the Ag alloy/glass interface. Raman peak positions were determined in two ways: (1) the wavenumber for which the maximum number of counts was detected, and (2) the peak position determined from fitting the peak to a Gaussian curve. Both procedures yielded the same value for the peak position. Peaks indexing, performed by comparing the positions of the peaks determined at the points (1) and (2) with data from literature for a phosphate glass [42], is summarized in the table embedded in Figure 8.

## 4. Conclusions

A jewel composed of a Nd-doped phosphate glass core surrounded by a silver alloy frame was successfully designed and fabricated. The glass was synthesized by conventional melt-quenching technique, whereas the metal frame was manufactured through the direct investment casting process followed by a burnout step. Ashby’s diagram reporting the melting point or the glass transition temperature versus the linear thermal expansion coefficient was built to be used as a guide for the selection of the most suitable metal alloy. The silver alloys revealed to be the best candidates for the fabrication of the jewel due to their enhanced thermo-compatibility with the glass. Among the broad compositional variety of silver-based alloys, the choice of an Ag-Cu-Ni-Zn alloy was due to its higher resistance to tarnish and to its superior mechanical properties in the as-cast condition, compared to traditional sterling silver. The thermo-mechanical properties of both the phosphate glass and the silver alloy were thoroughly investigated in view of identifying the optimal recipe for their successful joining. Despite the rapid heating to which the glass was subjected during the metal pouring, a solid jewel was successfully produced. No detrimental interaction was observed at the glass/metal interface, but only mechanical interlocking due to the metal filling the micropores at the glass surface. Due to the alloy chosen, it has not been necessary to heat treat the material after casting; besides the fact that a heat treatment would damage the glass, Ag-Cu-Ni-Zn alloy displays superior hardness than conventional sterling alloys already in the as-cast condition.

## Figures and Tables

**Table 1:** Ag-Cu-Ni-Zn alloy composition; each element concentration is expressed in (wt %).

Ag	Cu	Ni	Zn
95.70 ± 0.10	2.78 ± 0.07	0.70 ± 0.06	0.82 ± 0.07

**Table 2:** Nd^3+^ ions content in mol%, Nd^3+^ ions concentration, glass transition temperature (***T**_**g**_*), onset crystallization temperature (***T**_**x**_*), glass stability parameter (Δ***T***), glass softening point (***T**_**s**_*), coefficient of thermal expansion (CTE), density (***ρ***) and refractive index (***n***) at 633 nm of the ad-hoc synthesized Nd-doped phosphate glass.

**Nd^3+^ (mol%)**	1.2
**Nd^3+^ (** **× 10^20^ ions/cm^3^)**	1.6
***T_g_* (°C)** **± 3 °C**	508
***T_x_* (°C)** **± 3 °C**	791
***ΔT = T_x_ − T_g_* (°C)** **± 6 °C**	283
***T_s_* (°C)** **± 3 °C**	534
**CTE (** **× 10^-6^ °C^−1^)** **± 0.1 °C^−1^**	10.4
***ρ* (g/cm^3^)** **± 0.05 g/cm^3^**	3.32
***n*** **± 0.001**	1.585

**Table 3:** Alloy phases compositions measured via energy dispersive spectroscopy (EDS); each element concentration is expressed in (wt %).

Phase	Ag	Cu	Ni	Zn
Matrix	95.94 ± 0.26	3.03 ± 0.20	n.a.	1.03 ± 0.17
Eutectic	28.99 ± 0.51	60.79 ± 0.56	3.14 ± 0.26	7.07 ± 0.38
